# Trauma Dictations in the Emergency Department: A Quality Improvement/Patient Safety Project

**DOI:** 10.51894/001c.5126

**Published:** 2017-02-02

**Authors:** Eric W. Toth, Kristopher Richardson, Courtney Berry, Nikolai Butki

**Affiliations:** 1 Clinical Instructor/Emergency Medicine Resident, McLaren Oakland, Pontiac, MI; 2 Core Faculty, Emergency Medicine Residency Program, McLaren Oakland, Pontiac, MI; 3 Trauma Program Manager, McLaren Oakland, Pontiac, MI; 4 Associate Residency Program Director, Emergency Medicine, McLaren Oakland, Pontiac, MI; Assistant Professor, Osteopathic Medical Specialties, Michigan State University College of Osteopathic Medicine

**Keywords:** patient safety, dictation, trauma, emergency medicine

## Abstract

**CONTEXT:**

Trauma patients frequently represent a unique and challenging patient population in emergency medicine care settings. The policy of the McLaren Oakland Emergency Department (ED) is to have the treatment of all Level 1 and Level 2 trauma activations dictated by the ED resident. This policy is intended to facilitate both patient safety through clear communication between multiple medical services and quality improvement through reporting trauma specific quality metrics to third party agencies. Despite this requirement, trauma dictations in this setting were often found to not be completed. The purpose of this quality improvement/patient safety project was to implement a trauma dictation template to increase the rate of completed ED trauma dictations to above 75% of all Level 1 and Level 2 trauma activations.

**METHODS:**

A trauma dictation template was created to aid ED residents while completing trauma dictations. It was thought by the authors that the implementation of a standardized dictation template would help residents specify the necessary components needed to improve both patient safety and quality reporting. The development of the template was a collaborative effort between the emergency medicine residents and faculty, the trauma coordinator and trauma surgeons. The project was evaluated using two separate measures. A “process measure” was first used to determine if the addition of the trauma template made dictating less burdensome for ED residents, and an “outcomes measure” helped the authors examine whether template implementation actually led to an increased rate in completed trauma dictation.

**RESULTS:**

Data were collected during a three-month period prior to template implementation and three months after implementation. From November, 2015 through April, 2016, a total of 132 Trauma Activations were reviewed. The rate of completed dictations on Level 1 trauma activations increased from 45.16% to 53.33%. However, the rate of dictations on Level 2 trauma activations decreased from 50% to 30.4%, suggesting that Level 1 trauma patient care may have derived greater improvements from the new dictation template.

**CONCLUSIONS:**

The results of the ED residents’ evaluative survey responses were generally positive, indicating that the trauma template was perceived by most residents as a useful tool to complete dictations. Even though the outcome goal was not achieved, the project successfully achieved the goal of creating and implementing a usable trauma dictation template. Following the Plan-Do-Study-Act model for quality improvement/patient safety projects, the next project will examine additional barriers preventing users from utilizing this initial well-received tool.

## INTRODUCTION

Trauma patients often represent a uniquely challenging patient population in emergency medicine settings. Trauma patients frequently sustain injuries to multiple organ systems which necessitate consultation and treatment across multiple medical and surgical specialties. These consultations may occur across different locations quite distant from patients’ initial emergency department (ED) care setting. Therefore, patient safety principles necessitate accurate communications regarding patients’ initial presentation and ED care for all subsequent providers to convey correct and appropriate diagnostic and treatment plan information.^1^

As a Level II trauma center, McLaren Oakland has observed a trauma activation protocol since 2006. Using objective criteria concerning patients’ mechanism of trauma, physiology (i.e., vital signs) and anatomic findings (e.g., depressed skull fracture), trauma patients are assigned by providers into one of three trauma activation categories. Based on these criteria, the most critical and unstable patients are activated at a Level 1. Seriously injured but currently stable patients are activated as a Level 2. Stable patients are activated as a Level 3. Activating each injured ED patient into a trauma level initiates a chain of events and notifications designed to meet their anticipated clinical needs.^2,3^

For example, Level 2 activations automatically mobilize radiology and laboratory technicians to immediately present to the ED to obtain emergency radiographs and blood specimens. A Level 1 activation does the same, but also activates operating room and anesthesia personnel to anticipate and prepare for emergent surgery.

The current documentation platform used before this project in the McLaren Oakland ED in Pontiac, Michigan was a typical physician ‘T sheet’ template. These available paper templates are designed to be specific to the most common types of patients’ presenting chief complaints. Each template has a series of questions and fields concerning physical exam findings that are likely to be relevant to that chief complaint. The T sheet allows physicians to quickly document the key parts of an ED encounter by checking boxes and circling items for positive results, and crossing through negative finding fields.^3,4^

T sheets have long been used in EDs, and are widely used as excellent tools to capture the necessary elements of an encounter for billing purposes.^4^ Examples of T-sheets used for trauma cases include “Motor Vehicle Collision,” and “Multiple Trauma.”

Although efficient, T sheets tend to impose several limitations on providers. For example, they often provide very little space for clinician progress notes and documentation to inform providers’ subsequent medical decision making. The extensive use of circled boxes and pre-determined phrases on most T sheets also typically means that the individual thoughts of the physician are not recorded. It is therefore difficult for other providers to glean the critical details of the case when simply reviewing a T sheet.^3,4^

In addition, T sheets do not currently allow documentation of ATLS protocol elements for major trauma patients. There are generally no areas which designate primary and secondary survey information or a section for procedures, critical aspects of the management of most all trauma patients and outlined by both the American College of Surgeons (ACS), and the Advanced Trauma Life Support (ATLS).^2,5^ When it came to allowing providers to enter detailed documentation for their trauma patients, the standard physician T sheet is simply inadequate.

Since 2006, the McLaren Oakland Emergency Department had implemented a policy requiring that ED patient encounters leading to all Level 1 and Level 2 trauma activations be verbally dictated by providers in addition to their standard T sheet documentation. A verbal dictation to receiving providers was completed by the ED resident prior to the patient leaving the ED for the general medical floor. These dictations at the authors’ setting are transcribed by a medical transcription service with an average single business day turnaround time. These ED dictations are separate from the trauma History and Physical reports completed by the admitting medical services.

During this study, the purpose of the trauma dictation template was twofold: 1. To help facilitate patient safety by clearly communicating the patient presentation and ED treatment to multiple medical and surgical services subsequently caring for the patient, and 2. To improve the accuracy of quality trauma service metrics reported to third party agencies.

### Purpose

Despite the established trauma dictation policy, the authors noted that many Level 1 and Level 2 activations were not being verbally dictated. This quality improvement/patient safety (QIPS) project had two primary phases. The first phase was a systematic needs assessment to examine factors contributing to trauma dictations not being completed. The second phase included the creation and implementation of an easier streamlined template for trauma dictations. The overall goal of the project was to increase the percentage of completed Level 1 and Level 2 trauma activation dictation to over 75% during the six months after template implementation.

## METHODS

Since no patients were being studied during this project, a request for *Determination of* *Non-Human Subject Research* was completed in January, 2016 and approved by the McLaren Human Research institutional review board. During the design of the project, the authors had made extensive use of the *Plan, Do, Study, Act* (PDSA) sequential model for quality improvement as advocated by the Institute of Healthcare Improvement.^6,7^

The first step in the project was to identify barriers currently influencing the rates of complete trauma dictations made by EM residents. At the time of the study, almost 100% of all trauma patients were staffed by ED residents, making ED resident physicians the principal documenters and target group for the intervention. During September and October, 2015, three discussion groups were conducted, during which a group of over ten EM residents were asked why they thought trauma dictations were not being performed. The cause-and-effect or ‘fishbone’ diagram was used as a tool during these sessions to help determine **the** root causes of this problem.^8^ During the discussion groups, the three following primary barriers emerged: 1. Many EM residents were not aware that a dictation was required for all Level 1 and Level 2 trauma activations; 2. The dictation process was viewed by many participating residents as being prohibitively arduous and time consuming; and 3. It was unclear to some participants what information was expected to be included in the dictation.

In response to these perceived barriers, the next step in the project was to design a trauma dictation template based on published best practice examples.^9,10^ The template was designed through collaboration of the McLaren Oakland trauma surgeons, senior EM residents, the administrative trauma coordinator at McLaren Oakland and EM program faculty physicians.

The template (see Table 1) listed all of the information that the participating parties felt was necessary to achieve adequate levels of patient safety and included fields for information that the trauma coordinator needed for reporting quality metrics to payers. The template fit neatly onto a two-sided page and had been presented and approved at the Trauma Committee meeting in January 2016. After approval, the template was placed as a pdf file on the desktop of every computer in the ED department where verbal trauma dictations occurred.

**Table 1:** Trauma Dictation Template


**Trauma Dictation Template**
**Subjective**
· Age/ Gender
· Describe Mechanism: *“GSW to head”*
· CC: *“C/o of Headache”*
· HCC:
· PMH:
· PSH:
· Meds:
· Allergy:
· Social:

**Physical Exam**
Vitals: BP: RR: Pulse: Temp: O2Sat:

**Primary Survey** (*Relevant Negatives*)
· **A:** *Airway Patent, Patient in C- Collar placed by EMS*
· **B:** *Breath sounds equal B/L, breathing unlabored*
· **C:** *No active hemorrhage, Carotid, Radial, Femoral, Pedal pulses 2+ B/l.*
· **D:** *Pupils, GCS*
· **E:** *Full exposure*

**Resuscitation** – What was done to correct anything in the primary survey
· Intubated by…., Needle Decompression, Direct Pressure placed on hemorrhage, etc..
· Patient Placed on telemetry and continuous pulse ox.
· Two Large Bore IV placed
· Initial Fluid Bolus, Blood Products…

**Secondary Survey** (*Relevant Negatives*)
· Head: *No signs or trauma, laceration, abrasion*
· ENT: *No hemotympanum, No septal hematoma, Trachea Midline*
· Chest: *No crepitus, abrasion, bruising. RRR. Breath sounds clear B/L*
· Abdomen: *Soft. Not tender. No abrasion, bruising.*
· Pelvis: *Stable to rock. Not tender to palpation*
· Back: *Patient was log rolled. No deformity step-off or point tenderness*
· Extremities: *No obvious deformity, abrasion, laceration.*
· Neuro: *No focal lateralizing deficit. Cranial Nerves 2-12 grossly intact. Normal strength B/L. Normal sensation B/L.*
· GU: *No blood at urethral meatus, No scrotal hematoma, no perineal bruising. Rectal tone normal. No gross blood*


**Medical Decision Making**

**Trauma Activation**: What level, what criteria, by radio call or by presentation in ED

**Radiology**: Include what studies were ordered, why, and their results.
· eFAST EXAM and who performed it.
· Chest and Pelvis XR
· CT Head, C-Spine, Chest/Abdominal/Pelvis
· Other XR: Ankle, knee, wrist, etc...

**Procedures**: Central Line, Intubation, Chest tube, etc...Include who did it, and why it was done. Procedure note done separately

**Labs**:

**EKG**:

**Consultants**:
· Who did you talk to and why.
· Did they come in or by phone ( very important)
· What were their recommendations
· This includes the ICU resident.

**Progress Notes**: When did the surgical team arrived, when did the patient go to CT scan. When was a consultant paged. Describe the sequence of events as they occurred.
**Assessment**
· Include **ALL** diagnosis
· Laterality
· Don't forget small fractures, contusions, lab abnormalities, etc…

**Plan**
· Disposition ( Home, Admit, etc.)
· What Floor. Who are they admitted to.
· Pain Control
· PT/O T
· DVT/GI PPx
· Consultants for the floor
· C-Collar Cleared or not, and by whom
· Who did you transition care to (Surgical team, ICU team)

The final step in the project was an educational intervention designed to train the users of the new template. An educational lecture was delivered in January, 2016 as a 15-minute EM resident didactic session. The lecture was intended to educate the ED residents about the policy for completing dictations for all Level 1 and Level 2 trauma activation patients and how proper documentation of medical decision making was expected to impact ED patient care and safety. Short “booster” reminders of the dictation policy were provided later in the months following implementation during three subsequent three didactic sessions.

The impact of the project was evaluated using two separate measures. A “process measure” was first used to determine whether the availability and design of the dictation template was perceived to minimize perceived barriers and make dictating less burdensome for the EM residents. An “outcomes measure” also determined if the template actually led to an increase in the rates of completed trauma dictations.

To evaluate the process measure, a June, 2016 post-implementation survey was distributed to the EM resident users of the trauma dictation template. Answers were anonymous, with the intent to gauge the perceived usefulness of the dictation template among the residents. The residents were asked four questions regarding their awareness of the dictation template policy, existence and location of the template, whether they found the template useful, and whether they felt that dictating trauma cases affected the quality of the ED care they provided.

For the outcomes measure, a retrospective chart audit was completed to compare the percentage of completed Level 1 and Level 2 trauma activations with verbal dictations from the three months prior to the introduction of the template with the three post-implementation months.

## RESULTS

Project data concerning the outcome measure were collected by the authors’ healthcare system Trauma Department charged with quality control measurement. For this project, the primary metric was the rate of Level 1 and Level 2 trauma activations that had a dictation completed by the ED resident. No attempt was made to determine the quality of the dictation or evaluate any content in individual template dictations. Pre-implementation data were collected from November 2015 through January 2016, with post-implementation data extracted from February 2016 through April 2016. Overall, 132 trauma activations were reviewed during the total six-month project period.

During the three months preceding the template implementation, the complete dictation rate for Level 1 trauma activations were 14 out of 31, or 45.16%. Three months after the dictation template was introduced, the rate of complete Level 1 trauma activations had increased to 11 out of 21, or 53.33%. This 18.09% increase represented a minimal change from the pre-implementation rate, far below the authors’ goal rate of 75%. Of course, the number of trauma activations during this time period was largely out of the direct control of the investigators and the authors may certainly have been underpowered to demonstrate statistically significant pre-post differences using inferential statistical analyses.

**Figure 1:**
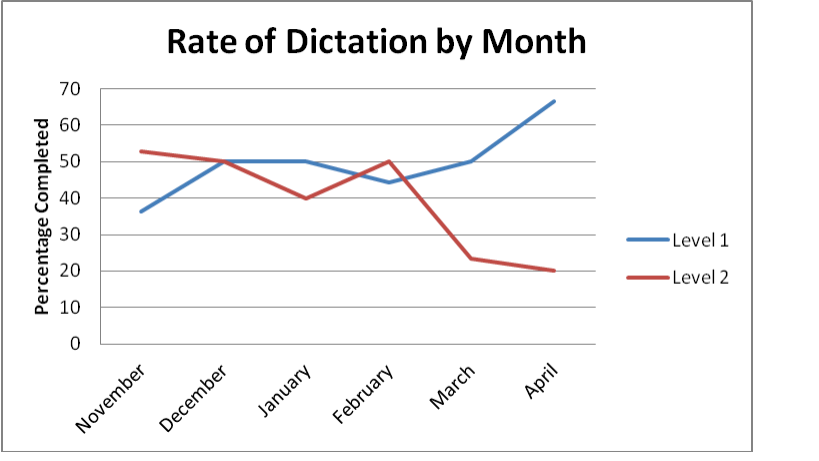
Changes in Complete Trauma Dictations after Template Implementation

The results for the Level 2 trauma activations demonstrated a decreased dictation rate after the introduction of the dictation template. In the three months before the dictation template implementation, the completed dictation rate was 17 out of 34, or 50%. After the introduction of the dictation template, the dictation rate actually decreased to 14 out of 46, or 30.4% (See Figure 1). This change represented a 39% decrease from the pre-implementation rate.

**Figure 2:**
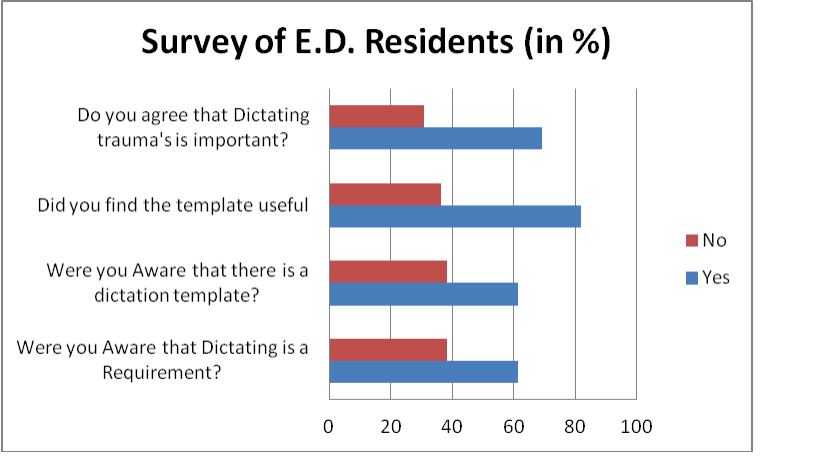
Survey of ED Residents regarding Template Use

To examine the selected process measure, a post-intervention survey was distributed to the EM residents regarding their experiences using the trauma dictation template. Overall, 13 (46.4%) of the 28 EM residents responded to the survey. Survey results indicated that although 8 (61%) of the 13 respondents were aware of the trauma dictation policy and that a dictation template was available, a large number 5 (39%) of 13 residents indicated that they were still unaware of the trauma dictation policy. Of those who had used the dictation template, 9 (81%) of 11 indicated that it was useful and made their trauma dictations easier. A majority 9 (69%) of 13 respondents expressed a belief that the dictation of trauma patients was important for patient safety and quality measurement and improvement (see Figure 2).

Anecdotally, the authors believe that the addition of the dictation template has had positive impact on the quality of their documentation of trauma patients. While not a primary measure of this project, several resident survey comments indicated that their trauma dictations were more thorough and organized after the implementation of the trauma dictation template. For example, one core EM faculty member commented that, “Now every injury and diagnosis is nicely documented, where before the diagnosis was often simply status/post Motor Vehicle Collision.” The McLaren Oakland Assistant Trauma Coordinator commented that, “When a trauma case has an ED dictated note, it is so much easier to tell what actually happened at two in the morning instead of just having to guess.”

## DISCUSSION

Judging from these results, the creation and implementation of the trauma dictation template may have alleviated some of the primary barriers perceived by ED residents to prevent them from completing trauma dictations. The survey results, however, also demonstrate that nearly 40% of respondents still remained ignorant of the trauma dictation policy requiring verbal dictation for Level 1 and Level 2 trauma activations after implementation. This finding suggests that lack of awareness remained a major barrier to meeting the goal dictation rate of 75%.

The authors learned several lessons during this QIPS project that could be used as next steps in the PDSA process. It appears that merely making a trauma dictation template available is not sufficient to increase the utilization of such a tool. Since the EM resident physicians in this setting were the principal documenters of trauma cases, increasing EM resident awareness of the template will prove critical to increasing its future use.

Providing a single educational session with subsequent ‘boosters’ during the same month was apparently insufficient for increasing some residents’ awareness of the new trauma dictation policy. The impetus for the next step in the PDSA cycle is that many residents were not at every didactic session every week. Many were either off service or absent from didactic sessions so as to comply with duty hour restrictions. Multi-month educational sessions may therefore be needed to capture more resident users.

The answers provided by those EM residents who did not favorably review the dictation template were also revealing. Several residents commented that they did not use the template because they had never before dictated a trauma despite being aware of the requirement. Most negative responses seemed to be directed toward questioning the perceived validity of dictation policies, not necessarily the elements of the process. Motivational techniques will also be considered as the next steps in the PDSA cycle to increase EM resident utilization of the dictation template.

### Limitations

A significant limitation of the study is the authors’ smaller sample size. With only 15-20 monthly Level 1 and Level 2 trauma activations occurring in this setting, the authors’ calculated dictation rate changes could have been inflated. The shorter period of intervention rollout for the project may have also limited the authors’ capability to note the full improvement derived from their dictation template. The post-intervention results demonstrating that 40% of the ED residents still denied having any knowledge of the dictation requirement should be considered during review of these results. It would also have been interesting to see what rate changes might have been over the course of an entire year. The authors believe the rate of completed trauma dictations would have likely increased as EM residents’ awareness of the template had risen.

One possible explanation for the decrease in Level 2 dictation rates was the time of year during which the project was conducted. The post-intervention months occurred near the end of the academic year, when the ED was increasingly staffed by more junior residents and off-service rotating residents. It is during this time of year when many of the junior ED residents now ATLS certified begin to staff trauma cases on their own. The senior residents are more likely to have been aware of the dictation requirement as they have more time to absorb the policies and procedures of the McLaren ED. Further education among new classes of EM residents will therefore need to be a permanent part of the annual Intern Orientation in July.

## CONCLUSION

Although the project did not achieve its outcome goal to increase Level 1 and Level 2 trauma dictation rates, the project was successful in that a trauma dictation template was developed and well-received by most users. There was also some anecdotal evidence that the quality of documentation for trauma patient care has been positively affected since the introduction of the template. The template also appears to have eased the perceived burden of documenting complex trauma cases with multiple injuries and consultants. By doing so, cross-provider communication has been improved. The next step of the PDSA cycle will be to further increase awareness and motivation to use the dictation template to ultimately increase rates of completed trauma dictations.

### Conflict of Interest

The authors have no financial or other conflict to interest disclosures to make.

## References

[ref-1911] Calleja P., Aitken L., Cooke M. (2016). Staff perceptions of best practice for information transfer about multitrauma patients on discharge from the emergency department: a focus group study. J Clin Nurs.

[ref-1912] Rotondo M.F. (2014). Verification, Review, Consultation Programs for Hospitals. Resources for Optimal Care of the Injured Patient.

[ref-1914] Bressan S., Franklin K.L., Jowett H.E., King S.K., Oakley E., Palmer C.S. (2015). Establishing a standard for assessing the appropriateness of trauma team activation: a retrospective evaluation of two outcome measures. Emerg Med J.

[ref-1915] Marill K.A., Gauharou E.S., Nelson B.K., Peterson M.A., Curtis R.L., Gonzalez M.R. (1999). Prospective, randomized trial of template-assisted versus undirected written recording of physician records in the emergency department. Ann Emerg Med.

[ref-1913] (2012). ATLS Student Course Manual: Advanced Trauma Life Support.

[ref-1916] Langley G.L., Moen R., Nolan K.M., Nolan T.W., Norman C.L., Provost L.P. (2009). The Improvement Guide: A Practical Approach to Enhancing Organizational Performance.

[ref-1917] Institute for Healthcare Improvement (2016). "Model for Improvement." How to Improve.

[ref-1918] Simon K. The Cause and Effect (a.k.a. Fishbone Diagram).

[ref-1919] Flaherty T. (2015). Templates Ease Documentation Burden, Respondents Say. HME News.

[ref-1920] United States Department of Health and Human Services (DHHS) (2013). Centers for Medicare and Medicaid Services (CMS). Pub 100-08 Medicare Program Integrity CMS Manual System.

